# American Indians and Alcohol

**Published:** 1998

**Authors:** Fred Beauvais

**Affiliations:** Fred Beauvais, Ph.D., is senior research scientist at the Tri-Ethnic Center for Prevention Research at Colorado State University, Fort Collins, Colorado

**Keywords:** Native American, Native Alaskan, prevalence, AOD use pattern, AOD associated consequences, history of AOD use, culturally sensitive prevention approach, AOD sales, comorbidity, AODD (alcohol and other drug dependence), behavioral and health disorder, causes of AODU (alcohol and other drug use), attitude toward AOD, expectancy, acculturation, sociocultural assimilation, treatment, public policy on AOD, federal government, literature review

## Abstract

The high prevalence of alcohol use and its consequences among American Indians may be attributed to a number of factors, including the influence of the European colonists who first made large amounts of alcohol available to Indians, as well as current social and cultural factors. Efforts to prevent and treat alcohol problems among the American Indian population may be more effective if native beliefs and approaches are incorporated. Alcohol problems also may be prevented through policies regulating the sale and use of alcohol in Indian communities.

Alcohol abuse and alcoholism have caused compounded problems for American Indian and Alaska Native peoples. In addition to the enormous physical and emotional tolls, the problems also have led to an unfortunate stereotype that has further burdened the Native communities of North America. This stereotype has perpetuated the image that all Indian people are afflicted with alcohol problems; even scientific inquiry, with its emphasis on problem definition, has not focused on the vast number of Indian people who maintain sober and productive lives (Beauvais in press). Furthermore, most studies of drinking among American Indians have focused on Indians living on reservations or on traditional Indian lands, even though this group accounts for only one-third of the American Indian population in the United States. With these caveats in mind, the following discussion outlines the historical and current status of alcohol use and abuse within the American Indian population, factors proposed to explain American Indian drinking and related problems, prevention and treatment approaches used in this community, and the role of alcohol policies in regulating American Indian drinking.

The nearly 2 million American Indians and Alaska Natives living in the United States fall into approximately 300 different tribal or language groups. Thus, although some generalities can be drawn with respect to alcohol use and its related consequences in this population, the variability among tribes and communities should not be disregarded. Alaska Natives are a diverse group themselves and have a somewhat different history and relationship to the U.S. Government than do other American Indians. Nonetheless, many similarities exist between them and the native people of the “lower 48” States with respect to alcohol use and abuse. Therefore, as a matter of convention, in this article the ethnic category “American Indian” also includes the indigenous people of Alaska. (For more information on alcohol use among Alaska Natives, see the sidebar by Segal, pp. 276–280.)

## Historical Context

Before European colonization, the native population of the territory that would eventually become the United States was relatively naïve to alcohol’s effects. Some tribes produced weak beers or other fermented beverages, but these were generally used only for ceremonial purposes. The distillation of more potent and thus more abusable forms of alcohol was unknown. When various European colonists suddenly made large amounts of distilled spirits and wine available to American Indians, the tribes had little time to develop social, legal, or moral guidelines to regulate alcohol use. Early traders quickly established a demand for alcohol by introducing it as a medium of trade, often using it in exchange for highly sought-after animal skins and other resources. Traders also found that providing free alcohol during trading sessions gave them a distinct advantage in their negotiations.

Extreme intoxication was common among the colonists and provided a powerful model for the social use of alcohol among the inexperienced Indian populations. Numerous historical accounts describe extremely violent bouts of drinking among Indian tribes during trading sessions and on other occasions, but at least as many accounts exist of similar behavior among the colonizing traders, military personnel, and civilians (Smart and Ogborne 1996). Such modeling was not limited to the early colonial era but continued as the land was colonized from East to West; trappers, miners, soldiers, and lumbermen were well known for their heavy drinking sprees.

History may have therefore sown the seeds for the prevalence of alcohol abuse in North American indigenous populations. Early demand, with no regulation and strong encouragement, may have contributed to a “tradition” of heavy alcohol use passed down from generation to generation, which has led to the current high level of alcohol-related problems.

## Contemporary Patterns of Alcohol Use

The level of alcohol use among American Indian adults is difficult to estimate. Drinking practices vary greatly from tribe to tribe as a result of cultural, economic, and lifestyle differences. Levy and Kunitz (1971) attributed the variability between tribes to differences in their tolerance of deviant behavior, which in turn lead to different levels of acceptable drinking. Other analysts have attributed the various rates to different socioeconomic conditions of reservations (Liban and Smart 1982; Silk-Walker et al. 1988; Austin et al. 1993). May (1996) reviewed the eight available studies on the prevalence of drinking among American Indian adults and found variation in the proportion of “current drinkers”^1^ from a low of 30 percent to a high of 84 percent; the rate among the general non-Indian population was 67 percent (May 1996). Hisnanick (1992) estimated the prevalence of alcohol abuse and alcoholism among American Indians using the number of patients discharged with an alcohol-related diagnosis from Indian Health Service (IHS) hospitals over an 8-year period. He found that northern reservations generally had much higher rates of such diagnoses (the highest rate was 111 per 1,000 population) than the southern reservations (the lowest rate was 11 per 1,000). Overall, the prevalence of an alcohol-related diagnosis among men was double the rate among women, a finding that is common in most surveys of Indian adult populations. In a review of existing data, May and Moran (1995), for instance, cited the rate of alcohol-related deaths for Indian men as 26.5 percent of all deaths and the rate for women as 13.2 percent. The gender disparity in consumption has not been seen among adolescents, however. Beauvais (1992) reported lifetime and 30-day prevalence among Indian adolescents to be only slightly higher for males than for females.

Compared with the limited available data on drinking by Indian adults, ongoing school-based surveys have provided a relatively complete picture of drinking among Indian youth since 1975 (Beauvais 1996). In 1993, 71 percent of Indian youth from grades 7 to 12 reported having ever used alcohol, and 55 percent reported having ever been drunk. Approximately 34 percent of this age group reported having been drunk within the past month. About the same proportion of Indian and non-Indian youth in grades 7 to 12 had ever tried alcohol in their lifetime. When Indian youth drank, however, they appeared to drink in heavier amounts and experience more negative consequences from their drinking than did their non-Indian peers (Oetting and Beauvais 1989).

Unlike the rates of illicit drug use, which tend to fluctuate over time, alcohol use among Indian youth has remained stable since 1975. Although tribal differences in drinking exist for adults, Indian adolescents seem to drink at similar levels regardless of tribe. In addition, higher levels of alcohol use have been found among Indian youth who live on reservations (Beauvais 1992), youth who attend boarding schools (Dick et al. 1993), and school dropouts (Beauvais et al. 1996).

Among both Indian and non-Indian adolescents, drug and alcohol use are much more tightly coupled than they are among adults. Nearly all adolescent drug users also use alcohol, and more than one-half of adolescent alcohol users use drugs at some level. It is likely that adolescent drug use and adolescent alcohol use have many of the same causes and consequences. Data from school surveys generally indicate that drug use is higher among Indian youth compared with non-Indian youth for nearly all drugs and that marijuana use in particular is significantly higher among Indian youth. In 1993, for instance, nearly 50 percent of Indian students in grades 7 to 12 admitted to having ever used marijuana (Beauvais 1996), whereas the rate for non-Indian youth of the same age was just 12 percent (Substance Abuse and Mental Health Services Administration 1994).

Many researchers (Mail and McDonald 1980; May 1996) have reported a style of drinking frequently engaged in by both Indian youth and adults in which drinkers consume large amounts of alcohol in a short period of time and continue drinking until the supply is gone. This pattern—consuming five or more drinks in one session—is often called “binge drinking.” Furthermore, notable sharing of alcohol takes place among people drinking together. This pattern has been attributed to the early modeling of European colonists previously mentioned as well as to the effects of prohibition, which encouraged rapid drinking to avoid the detection and confiscation of alcohol. This style of drinking is only one of a wide range of styles practiced within Indian communities; aside from the research conducted by May (1995), however, little has been done to characterize the various patterns.

Building on the work of Ferguson (1968), May (1995) proposed that at least two patterns of alcohol abuse exist within Indian groups. “Anxiety drinkers” are chronic, heavy drinkers who exhibit a wide variety of medical, social, and psychological problems. They have minimal involvement in their traditional Indian culture and show little competency in meeting the demands of the majority culture (e.g., maintaining employment). Early alcohol-related mortality is common among this group.

In contrast, “recreational drinkers” engage in binge drinking less frequently than anxiety drinkers do, but they consume extremely high quantities when they do drink. (Although May termed this style of drinking “recreational drinking,” other researchers and treatment professionals have called it “problem,” “binge,” and “heavy episodic” drinking.) Although when not drinking, recreational drinkers can function fairly well in other areas of their lives, they do experience many alcohol-related consequences, with accidents of all types being the most common. Proportionally, recreational drinkers are the largest group of Indian alcohol abusers (about two-thirds of all heavy drinkers according to May [1996]) and thus account for the largest number of alcohol-related problems in Indian communities.

Beauvais (1992) reported similar drinking patterns among Indian adolescents. According to his research, some Indian youth become heavily involved with alcohol and illicit drugs at an early age and continue that pattern into at least young adulthood. Approximately 20 percent of Indian youth in 7th to 12th grades fall into this category. Other youth exhibit an “experimental” or social style of use that varies with different environmental conditions and does not necessarily lead to a lifelong alcohol abuse pattern. Beauvais (1992) reports that another 20 percent of Indian youth are in this group.

## Consequences of Alcohol Use and Abuse

Compared with the U.S. population in general, the American Indian population is especially at risk for alcohol-related consequences. According to IHS records on alcohol-related illness and death among tribes in the United States (IHS 1996), the age-adjusted alcohol-related death rate in 1992 was 5.6 times higher among the Indian population than among the U.S. population in general; this rate was 7.1 times higher in 1980. Nearly twice as many Indian men as Indian women die from alcohol-related causes between ages 45 and 64, the peak age range for such deaths. Chronic liver disease and cirrhosis are 3.9 times as prevalent in the Indian population as in the general population; alcohol-related fatal automobile accidents are 3 times as prevalent; alcohol-related suicide is 1.4 times as prevalent; and alcohol-related homicide is 2.4 times as prevalent.

The data clearly demonstrate that the health consequences of alcohol abuse have a much greater effect on the Indian population than on the non-Indian population. The ratio of drinkers to abstainers in Indian and non-Indian populations is not well documented, however. May (1995) suggested that a greater percentage of Indian adults may abstain from alcohol compared with non-Indian adults. Therefore, the higher levels of problems within the Indian population may indicate that those who do use alcohol drink at exceptionally high levels.

High rates of fetal alcohol syndrome (FAS) have been found among some Indian tribes. May (1991) found rates ranging from 1.6 to 10.3 births per 1,000 for a selected number of tribes. The rate among the general, non-Indian population is 2.2 births per 1,000. Because of the high FAS prevalence rates and because FAS is an entirely preventable cause of birth defects, several Federal and tribal agencies have given a high priority to FAS research and prevention efforts.

## Comorbidity of Alcohol Problems and Mental Disorders

Alcohol and drug abuse problems often are attributed to underlying psychological disorders; consequently, those disorders have been cited as contributing to alcohol problems among American Indians (Mail and McDonald 1980; Novins et al. 1996). Theoretically, people with psychological problems use alcohol to relieve certain symptoms, such as depression, anxiety, or lack of self-esteem. In response to this theory, many alcohol prevention and treatment programs address those psychological problems. The literature for both Indian and non-Indian populations, however, clearly indicates that psychological or emotional problems and substance use are not necessarily related, particularly among adolescents (Oetting et al. 1998; Schroeder et al. 1993; Swaim et al. 1989; Beauvais 1992). The link between psychological problems and alcohol abuse may be stronger among adults than among adolescents.

Although not necessarily a cause or consequence of alcohol abuse or alcoholism, other mental disorders often co-occur with alcohol disorders. No studies have compared the prevalence of co-occurring psychiatric disorders between Indians and non-Indians, but clearly such disorders are common among Indian populations. Robin and colleagues (1998) found that among a large group of adults from a Southwestern tribe, binge drinkers were 5.5 times more likely to have had a psychiatric problem than nondrinkers. In a study of adolescents on a Northern Plains reservation, Beals and colleagues (1997) reported that more than one-half of the youth diagnosed with a psychiatric disorder also had a substance use disorder. Caution must be used in generalizing from those data, however, because they are derived from clinical populations and may not represent the majority of youth. As discussed earlier, different types of alcohol users exist, and mental disorders may only occur among heavy drinkers. Among adolescents in particular, considerable “experimental” drinking likely occurs in the absence of any psychiatric problem and probably is more social in nature (Oetting et al. 1998; Austin et al. 1993). Therefore, programs to reduce alcohol consumption that presume that all drinkers have an emotional problem probably will be ineffective with the largest group of alcohol users.

## Causal Explanations for Alcohol Abuse and Alcoholism

The history of alcohol use among Indian tribes, as described earlier, sets the stage for the high rate of alcohol-related consequences currently reported for this population. A number of more contemporary factors have been proposed to explain the continuation of this pattern.

The following sections describe some of those factors, including genetics, social and cultural influences, and personal attitudes toward alcohol.

### Genetic Risk Factors

Evidence for a genetic component in the susceptibility to alcoholism has been increasing over the past three decades. Kendler and colleagues (1997) estimated that among males, genetic factors account for 50 to 60 percent of the risk for alcoholism. Evidence of a genetic component to alcoholism raises the question of whether certain ethnic and cultural groups that have high rates of alcoholism, such as American Indians, may be predisposed to higher alcohol consumption. Research has identified differences among population groups in the enzyme systems that regulate alcohol metabolism; those differences are thought to account for some cultural differences in drinking patterns. For instance, among many Asian groups the absence of certain metabolic enzymes results in the “flushing response,” an unpleasant reddening or flushing of the skin, sometimes accompanied by nausea, after drinking alcohol. The discomfort that accompanies the flushing response is credited with fostering lower levels of alcohol abuse in some Asian populations. If a genetic factor could be identified that explained the high rates of alcoholism among American Indians, prevention and treatment strategies could take that factor into account and perhaps thereby improve their effectiveness (e.g., through genetic counseling or the development of drug treatments to alter the genetic effects). So far, the evidence seems to indicate that although some proportion of alcoholism risk may be heritable, this trait varies more within population groups than between them. In other words, certain groups may not be more susceptible to alcoholism, but a proportion of persons within groups might be. Furthermore, the search for genetic susceptibility among groups of American Indians may be hampered by the increasing amount of intermarriage and childbearing both within Indian populations and between Indians and non-Indians, thus obscuring any genetic component that might be present (Snipp 1997).

Although identification of the genetic factors that contribute to alcoholism may aid in our understanding of the risk for alcoholism, identifying these factors may not help reduce alcoholism among populations where it is most prevalent. Other influences, such as social and cultural factors, are at least as potent, and possibly more potent, than genetics in the development of alcoholism. Addressing those environmental factors likely will have a higher long-term payoff.

### Social and Cultural Influences

#### Socioeconomic Factors

The socioeconomic picture for many tribes is bleak. Unemployment rates are high, school completion rates are low, and basic support systems are underdeveloped. Those conditions place a great deal of stress on the family and other socialization structures within Indian communities. As a result, the basic developmental needs of Indian children often go unmet. To the extent that this type of social stress predisposes a population to alcohol abuse, American Indian communities are highly susceptible.

#### Boarding School Experience

Until recently, most Indian children were removed from their homes (sometimes forcibly, by social service agencies) and placed in boarding schools that were often hundreds of miles from their families. Some children would not see their families for months or even years. The conditions at the boarding schools were quite severe, and behavior was shaped primarily through punishment. Both physical and emotional abuses were common. In addition to their traumatic effects on children, these abusive practices spawned several generations of Indian people with limited parenting experience. Children raised in boarding schools often perpetuated the schools’ punitive model later with their own children. Parents did not have the opportunity to raise their children in a way that was culturally congruent. In fact, the intent of the boarding schools was to eliminate Indian culture and replace it with white culture. That practice led to an accelerated weakening of the values, beliefs, and cultural forms that had previously guided behavior in Indian communities.

#### Loss of Culture

Many Indian people believe that the loss of their culture is the primary cause of many of their existing social problems, especially those associated with alcohol. Many of the community-based alcohol treatment programs in Indian communities across the country have a strong cultural or spiritual component that is intended to revitalize traditional beliefs and serve as the primary source of individual strength in maintaining sobriety. The research community, however, has been reluctant to accept the idea that culture and Indian spirituality may be important to the prevention and treatment of alcohol problems. At least two reasons exist for this attitude. First, non-Indian views of the psychology of behavior are primarily secular and, for the most part, relegate culture to a peripheral role. Second, methods to measure spirituality, cultural beliefs, and values have not been well developed, hindering scientific study in those areas. A number of recent studies have attempted to find a link between cultural identification and substance use among Indian adolescents, but so far no relationship has been found (Beauvais 1998). Although culture may not be a protective factor, at least for Indian adolescents, it also may not have been properly characterized and thus not accurately measured. The extremely strong belief, held by Indian elders and others, that culture is a critical protective factor suggests that more research is needed in this area.

#### Cultural Forms

Some researchers have suggested that current Indian drinking may be a product of the early ceremonial use of alcohol (Abbott 1996). Before colonization, however, few tribes had access to any alcohol—certainly not to distilled spirits. The extent of the current problem thus cannot be linked to longstanding cultural practices. Indian drinking has been associated with the search for transcendental experiences, and some authors (Kahn 1986; Mail and McDonald 1980) have drawn parallels with “vision quests” and other cultural rituals that are purported to put one in contact with supernatural forces. Colonial-era writing by Indians recounting their perceptions of the subjective effects of alcohol when it was first introduced lends some credence to this view. However, a comparison between current drinking patterns and the ceremonial use of alcohol reveals many differences. Rituals that are intended to produce visionary experiences are highly controlled and leave little room for aberrant or disruptive behavior. The peyote ritual is a prime example. Within the Native American church, peyote is a sacrament intended to put one in communication with spiritual forces to instill harmony in one’s life. The ceremony itself has a prescribed structure, and the leader, called a “roadman,” makes certain that the rules and forms are precisely followed. Peyote is used to facilitate communication and is not ingested to produce hallucinations. The roadman will use a variety of sensory stimuli (e.g., cedar smoke and sprinkled water) to prevent participants from drifting off into a disconnected state of consciousness. Clearly, the powerful and ritualized experience of a peyote ceremony cannot be likened to out-of-control drunkenness. In fact, the peyote ritual is often used within Indian communities for the treatment of alcohol and drug abuse problems.

### Attitudes and Expectancies

American Indians appear to vary somewhat in their perceptions of alcohol and its effects. Spicer (1997) reports that most Indians who drink are ambivalent about alcohol. On the one hand, they view drinking as a social mechanism that facilitates interactions with family and friends and increases bonding; on the other hand, alcohol abusers are acutely aware of the destruction it has wrought in their lives. A tendency also exists for Indian drinkers to believe that Indian people have a special susceptibility to the effects of alcohol, both from physical vulnerability and from “being Indian” (Mail and McDonald 1980). Some writers have speculated that alcohol abuse has become an identifying trait among American Indians and that sobriety often results in people being disenfranchised from their social milieu (Mail and McDonald 1980; Spicer 1997). Social factors thus strongly influence the use of alcohol in this population.

Cultural beliefs among many Indian tribes place responsibility for behavior outside the person and in the realm of spiritual forces, both good and evil. According to these beliefs, the resolution to a particular problem lies in the ceremonial realm, in which a person remains relatively passive while rituals are performed to resolve the imbalance of powers. This approach is distinctly different from the Western European notion that each person is ultimately responsible for his or her own behavior and that change comes only through personal initiative or with assistance, such as psychotherapy. One belief is not superior to the other; however, the Indian perspective (as with any perspective) could be distorted in an effort to justify and continue one’s harmful behavior. One can easily say “it is out of my hands,” continue drinking, and not seek the ceremonial assistance needed to achieve sobriety.

## Prevention and Treatment

Understanding the factors that contribute to the high rate of alcohol-related problems in the Indian population is helpful in developing prevention and treatment strategies. Many tribes recognized the need for the prevention of alcohol abuse soon after alcohol problems first began to appear. The appeal of alcohol was not universal among tribes, and numerous efforts were made to maintain or regain sobriety. Many tribes attempted to restrict commerce in alcohol and were successful for short periods of time (Smart and Ogborne 1996). The Handsome Lake movement of the early 1800s, named for the Seneca religious leader who incorporated a vigorous antialcohol stance into traditional beliefs and ceremonies, was the most notable early attempt to prevent alcohol problems through the use of cultural practices (Jilek 1978).

In recent decades, prevention of alcohol abuse has been a high priority within American Indian communities. Community leaders, school personnel, and health providers have recognized the toll that alcohol is taking and have instituted a variety of prevention interventions. The IHS, the Bureau of Indian Affairs (BIA), numerous Federal research and service organizations, and tribes themselves have been involved with and have provided resources for prevention. Unfortunately, most of those efforts have not been rigorously evaluated. Although some interventions may help lead to lower rates of alcohol use, it is difficult to determine which approaches are the most effective.

The IHS has provided treatment for alcohol abuse and alcoholism since its inception in 1975. In addition to numerous tribally based programs, the agency currently funds 7 regional treatment facilities for women and 12 for adolescents. In the past decade, much of the central responsibility for running those programs has shifted from the Federal Government and the IHS to tribal control. Accompanying the trend toward tribal control is a movement toward the use of traditional cultural and spiritual beliefs and practices in treatment. In some cases non-Native approaches, such as detoxification, pharmacotherapy, behavioral therapy, inpatient treatment, and Alcoholics Anonymous, have been modified to incorporate Indian beliefs and traditions. Sweat lodge ceremonies, the peyote ceremony, smudging with smoke, and traditional dancing and singing (Jilek 1978, 1994; Manson et al. 1987) are increasingly incorporated into Indian treatment programs. Unfortunately, no randomized trials or other controlled studies have been conducted to test the efficacy of those efforts.

### Alcohol-Related Policies

Prohibition has been the most prevalent policy in attempting to reduce alcohol consumption among Indian tribes, although it has been inconsistently applied. In colonial times, tribes as well as non-Indian authorities attempted to limit the sale or importation of alcohol within Indian territories. The bans were mostly ineffective, however, because alcohol was involved in lucrative trading and someone was always willing to distribute it. Alcohol even became a political issue when the British and French governments vied for the “friendship” of various tribes by providing alcohol (Smart and Ogborne 1996). In 1832 the U.S. Congress passed legislation banning the sale of alcoholic beverages to Indian people. That legislation was repealed in 1953, and tribes were given the option of retaining prohibition or allowing the sale and consumption of alcohol on reservations. Today nearly two-thirds of all reservations are technically “dry.” Little is known about the effects of the Federal legislation before 1953, although most observers would agree that it was not very effective. May (1992) and Bellamy (1985) examined the effects of the then-current prohibition laws by comparing “wet” and “dry” reservations with a number of factors, such as health indices and accident rates. For the most part, the researchers found few differences between wet and dry reservations. However, May raised the issue of whether prohibition actually created more problems, because people who went off the reservation to drink were more susceptible to death and injury from exposure and from driving under the influence.

Reflecting on the weak results of prohibition, May (1992) suggested that legislation alone is not the answer. He called for a community consensus to be developed regarding the use of alcohol as well as a comprehensive approach to involve multiple community agencies and groups. In lieu of such a consensus, it would be impossible for any one responsible community faction to “enforce” a common standard.

Policies regulating the sale and use of alcohol can serve as important tools in preventing alcohol problems and merit increased attention among tribes. However, although such policies may succeed to some extent in community settings such as reservations, they may be more difficult to implement in urban or rural settings in which American Indians are only a small portion of the total population. Currently, many different agencies implement alcohol policies and claim some responsibility for lowering the rates of alcohol use. As a result, policies are inconsistent, contributing to uncertainty in the Indian community, especially among adolescents, about normative use and sanctions against illegal use.

The potential for regulation through policy is substantial. May (1992) listed 107 policy options that could be considered by tribes to control levels of use within communities. The options were divided into the categories of controlling supply, shaping drinking practices, and reducing social and physical harm. All options are not feasible everywhere, but a core set could be implemented in most communities. A strong research initiative to evaluate the effects of policy implementation and changes also seems warranted. Certainly, policy alone is not the only route to prevention, because it does not address the powerful factors leading to alcohol abuse. Consistent policy, however, can serve as an important message of what the community as a whole considers both acceptable and unacceptable behavior.

## Conclusion

American Indian and Alaska Native communities experience high rates of alcohol-related problems and have responded by implementing prevention and treatment programs, including both grassroots and externally sponsored programs. Perhaps the greatest impetus for change regarding alcohol use in Indian communities has been the revitalization of Indian culture, which began during the 1960s Civil Rights Movement. The Federal Government is gradually reducing its caretaker role, and tribes are assuming greater authority over their own economic, social, educational, and health affairs. Furthermore, awareness is growing that solutions to social and health problems must be generated at the community level and those that have been imposed from outside will most likely be ineffective (Beauvais and LaBoueff 1985; Oetting et al. 1995). Perhaps the most powerful and effective solutions will come through a recommitment to traditional Indian values and beliefs. Combined with a concerted and consistent message from the many social support systems in Indian communities, that approach will, one hopes, lead to a substantial reduction in alcohol-related problems.

Little research-based data exist about the factors that lead many, if not most, Indian people to remain sober or to regain their sobriety and lead fulfilling lives. A great number of Indian people can drink socially and not incur serious problems. If more information could be gained about those groups of people, that knowledge could be applied to efforts to prevent alcohol abuse and alcoholism in the Native American population. Research on alcohol problems among urban Indians also would be useful, because it would improve understanding of how contextual social variables affect the course of alcohol abuse.

## Footnotes

1 In general, current drinkers are those who have consumed alcohol within the past year, but different researchers use different definitions. In this article, drinking categories refer to the definitions used in the research cited.
